# Detection of Hazelnut and Almond Adulteration in Olive Oil: An Approach by qPCR

**DOI:** 10.3390/molecules28104248

**Published:** 2023-05-22

**Authors:** Sonia Ramos-Gómez, María D. Busto, Natividad Ortega

**Affiliations:** Department of Biotechnology and Food Science, Area of Biochemistry and Molecular Biology, University of Burgos, Plaza Misael Bañuelos s/n, 09001 Burgos, Spain; soniarg@ubu.es (S.R.-G.); dbusto@ubu.es (M.D.B.)

**Keywords:** authenticity, *Corylus avellana*, melting curve analysis, *Olea europaea*, olive oil adulteration, *Prunus dulcis*, qPCR

## Abstract

Virgin olive oil (VOO), characterized by its unique aroma, flavor, and health benefits, is subject to adulteration with the addition of oils obtained from other edible species. The consumption of adulterated olive oil with nut species, such as hazelnut or almond, leads to health and safety issues for consumers, due to their high allergenic potential. To detect almond and hazelnut in olive oil, several amplification systems have been analyzed by qPCR assay with a SYBR Green post-PCR melting curve analysis. The systems selected were Cora1F2/R2 and Madl, targeting the genes coding the allergenic protein *Cor a 1* (hazelnut) and *Pru av 1* (almond), respectively. These primers revealed adequate specificity for each of the targeted species. In addition, the result obtained demonstrated that this methodology can be used to detect olive oil adulteration with up to 5% of hazelnut or almond oil by a single qPCR assay, and with a level as low as 2.5% by a nested-qPCR assay. Thus, the present research has shown that the SYBR-based qPCR assay can be a rapid, precise, and accurate method to detect adulteration in olive oil.

## 1. Introduction

Virgin olive oil (VOO) is obtained from the fruit of the olive tree (*Olea europaea* L.) by mechanical or other physical means only under conditions, particularly thermal conditions, which do not lead to the alteration of the oil, and which have not undergone any treatment other than washing, decantation, centrifugation, and filtration [1]. VOO, one of the key foods of the Mediterranean diet, is valued for its sensory, nutritional, and health characteristics, compared to other vegetable oils [2]. The leading producer, consumer, and exporter of olive oil in the world is Europe, with a consumption share of about 66% [3]. In the 2019–2020 season, the world olive oil production reached 3.5 tons, and 60% was produced in the European Union (EU). In terms of production, 91.8% of EU olive oil production (about 1.8 million tons) is produced by three Mediterranean countries: Spain (58.48%), Italy (19.02%), and Greece (14.29%) [4].

The high commercial value of the VOO makes it susceptible to deliberate adulteration with other low-priced edible oils. In addition to being a fraud for the consumer, who pays for a quality product, it can cause serious health problems if vegetable oils from potentially allergenic species such as hazelnut (*Corylus avellana*) or almond (*Prunus dulcis*) are added. The detection of adulteration of olive oil with most other vegetable oils can be carried out using conventional methods based on the differences in the triacylglycerol, fatty acid, or sterol composition of these oils [4,5]. Thus, fatty acid composition can be used to detect olive oil adulteration with canola, soybean, rapeseed, walnut, mustard, and peanut, with a level below 5% [6,7,8,9,10]. The absolute value of the difference between the experimental and theorical equivalent carbon number 42 (ECN42) in triacylglycerols can be used to detect, at levels as low as 1%, olive oil adulteration with sunflower, cotton, safflower, soybean, rapeseed, corn, canola, sesame, and walnut [7,11]. Based on differences in triglyceride and fatty acid composition [7], olive oil adulteration with canola, sesame, soybean, cotton, sunflower, corn, safflower, and walnut can also be detected.

Hazelnut oil is one of the most common adulterants found in olive oil due to the very similar chemical profiles (triacylglycerol, total sterol, and fatty acid composition) of these two oils [12]. This adulteration has been estimated to cause a loss of four million euros per year for countries in the European Union [13]. Conventional methods used for other vegetable oils do not detect the adulteration of olive oil with hazelnut oil at low concentration levels (5–20%) [14]. Therefore, there is a great interest in the development of analytical methods to detect olive oil adulteration, and to guarantee the authenticity and traceability of virgin olive. Several chromatographic and spectroscopic methods have been suggested in order to detect the presence of hazelnut oil in olive oil: SPME-GC/MS (solid phase microextraction-gas chromatography/mass spectrometry) analysis [15], FT-MIR (Fourier-transform mid-infrared) spectroscopy [16,17], FT-IR (Fourier-transform infrared) spectroscopy coupled with chemometrics [18], fluorescence spectroscopy [19], Raman spectroscopy [17,20], proton NMR (nuclear magnetic resonance) alone [21,22,23,24] or combined with ^31^P NMR [25,26] and ^13^C NMR [27,28].

Almond oil is also used in the food industry nowadays, although not as common as VOO, but is also used in salads and vegetable dips, and its composition makes it a healthy alternative given its high levels of vitamin E and unsaturated fatty acids [29]. Despite its food interest, it has been described as an olive oil adulterant [30] and has been included in several studies on olive oil adulteration [2,16,25,31]. The main problem of a potential adulteration of olive oil with almond oil lies in the possible allergic reactions to some of the compounds found in these edible oils [32] and it is therefore essential for consumers to know the quality, composition, and nutritional information of the products they are buying. Almond allergy also deserves special attention because low doses can induce severe allergic reactions [30]. Although almond detection has been little studied in vegetable oils, various detection methods have been described, many of them relying on immunochemical [33,34], mass spectrometry [35], visible spectroscopy coupled with artificial neural network [2], and DNA-based [36] techniques. Aroca-Santos et al. [2] reported the quantification of sweet almond oils in ternary mixtures with extra virgin olive by visible spectroscopy with linear regressions or MLP (multi-layer perceptron).

In recent years, the use of DNA-based techniques has become important in testing the authenticity of olive oil due to its high sensitivity, specificity, and reliability [6,37]. DNA analysis detects differences at the genome level, so the similarity between the chemical composition of olive oil and its adulterants is not a problem for fraud detection with these methods. The molecular markers such as the microsatellites or single sequence repeats (SSRs) and the single nucleotide polymorphisms (SNPs) are used for olive oil traceability purposes [38,39,40]. Furthermore, these markers can provide information about the varietal origin of olive oil or the botanical (species) origin of plant oils, and therefore allow the detection of any adulteration in olive oil. Polymorphisms are genetic variants that can be detected both in the nuclear genome and in genomes associated with organelles such as the chloroplast [41].

In the last decade, qPCR (quantitative polymerase chain reaction) has been used in the authentication of edible oils [42,43,44,45]. This technique, characterized by greater sensitivity, reliability, and specificity than conventional PCR, allows the detection and quantification of DNA in very low amounts. In addition, qPCR molecular markers, that require short-length PCR amplification, are suitable as analytical targets in partially degraded DNA samples, as those of olive oil [45]. qPCR has been applied in the detection and authentication of VOO. Ganopoulos et al. [46] and Vietina et al. [47] proposed the high-resolution melting analysis (HRM) of *rbcL* gene (ribulose-1,5-bisphosphate carboxylase/oxygenase) amplicons to detect adulteration of olive oil with corn, sunflower, hazelnut, and canola oils. Ramos-Gómez et al. [48] reported that the PetN-PbM system, based on the region between the *PetN* gene (encoding a cytochrome b6/f complex subunit N) and *PsbM* gene (encoding a photosystem II protein M), showed to be a suitable and reliable system in relation to olive oil and olive ingredients in both food authentication and food safety processes. A qPCR method based on the chloroplast *trnL* (UAA) gene was used to establish a method for the authentication of olive oil [45]. Uncu et al. [49] reported that while fatty acid analysis did not detect fraud adulteration of olive oil with hazelnut oil, the presence of this adulterant at a relative content as low as 5% was successfully determined with a chloroplast *trnL* (UAA) intron gene PCR-CE (capillary electrophoresis) method [49].

In this context, the main objective of this work consists of testing the capability of the real-time PCR assay with a SYBR Green post-PCR melting curve analysis to provide a fast, reliable, and cost-effective method for the detection of the adulteration of VOO with hazelnut or almond oil at low percentages.

## 2. Results and Discussion

### 2.1. DNA Extraction

The reliability of the results obtained by qPCR depends largely on the selection of an optimal DNA extraction method for the species under study and the starting matrix. In the case of leaf tissue, numerous optimized protocols are available for different species, but the high fat and polyphenol content of olive, hazelnut, and almond leaves can interfere with the DNA isolation. Thus, in this work, one of the most widespread CTAB-based methods for DNA extraction from leaves described by Doyle and Doyle [50] was compared with an adaptation of this method for leaf tissues with high lipid and phenolic contents [45].

The spectrophotometric results showed that, both in quality and quantity, the adapted protocol yielded better results. Specifically, the concentration of DNA extracted with the adapted method was approximately three times higher than that obtained with the classical protocol for leaf tissue of hazelnut (206.1 ± 17.2 versus 591.5 ± 4.0 ng/µL) and almond (173.7 ± 19.1 versus 548.3 ± 42.4 ng/µL). In the case of DNA obtained from olive leaves, the yield was much higher, from 25.7 ± 1.6 ng/µL (classical method) to 574.3 ± 14.3 ng/µL (adapted method). Moreover, the use of the adapted method also improved the purity ratios, reaching A260/A280 values of 1.58 ± 0.05, 1.71 ± 0.07, and 1.81 ± 0.06 for DNA from almond, hazelnut, and olive, respectively.

The low purity ratios obtained from leaves of these species were similar to those previously reported in other studies [51]. These highlight the difficulty of achieving the optimal ratio of 1.8 but suggest that DNA obtained at these ratios is amplifiable [52]. In fact, the use of commercial kits by other authors showed the difficulty of finding a kit adapted to the characteristics of this type of tissue, even failing to obtain amplifiable DNA [53], finding optimal results in protocols based on CTAB with modifications [53,54,55].

The extraction of DNA from vegetal oils was carried out by the protocol described by Ramos-Gómez et al. [44]. This method is based on the use of chloroform, as an organic reagent, using phenol:chloroform:isoamyl alcohol (25:24:1) in a volume equal to the volume of olive oil, and avoiding several stages of DNA precipitation. The DNA concentration obtained for olive, almond, and hazelnut oils was 941 ± 14, 5.55 ± 0.62, and 1.77 ± 0.81 ng/µL, respectively. The amount of DNA recovered from vegetable oils by any of the described methods is too small, below 15 ng/μL [56], to be detectable by either agarose gel electrophoresis or UV spectrophotometry [51], with the exception of Raieta et al. [57] who were able to visualize DNA obtained by a CTAB-based method and purified by electro-elution using agarose gel electrophoresis. In terms of quality, all samples had similar values of A260/A280 ratio (1.179 ± 0.269). Similar low absorbance ratios have been detected in DNA isolated from vegetable oil in previous works, indicating the presence of protein, phenol, or other contaminants [44], but they also reported the amplifiability of these samples.

### 2.2. Selection of a Specific System to Detect Almond or Hazelnut

The detection of hazelnut and almond was performed using primer sets obtained from literature, which are based on main targets in hazelnut and almond specific detection systems. The genes considered as potential targets in the development of hazelnut-specific systems for application in the agri-food sector mainly involve the gene *Hsp1* coding for low molecular weight heat-shock protein and the genes coding for the Cor, a group of allergenic proteins. In this regard, two systems were selected based on *Hsp1*: Hsp1 [58] and Nocc1 [59]. The Hsp1 showed specificity against 28 species, and the Nocc1 system against 17 species. The amplification systems based on the *Cor a* gene selected were: Cora1FW/RS [60], Cora1F2/R2 [54], and Cora13 [53]. Cora1FW/RS was a specific hazelnut system tested against 13 species and some commercial foodstuffs; Cora1F2/R2 amplified, in different food matrices, only hazelnut among other 15 species; and Cora13 allowed a specific amplification of hazelnut against seven species in processed food. The specificity of these systems against olive has not previously been checked, and they have not been used to analyze DNA from vegetable oils.

The selected amplification systems for detecting almonds were Madl [61], Prd6 [58], Pru du 1 [36], AlmondITS [62], and thau [63]. Madl is based on gene *Pru av 1*, for major allergen PRU, and showed specificity against 44 species, detecting cross-reactivity only with apricot. Prd6 was designed on gene encoding for the Pru du 6 allergen and has been tested on 12 almond cultivars and other 26 plant species. Pru du 1 was also designed on the gene coding for major allergen protein Pru du 1 and has been analyzed with seven species and nine commercial products. The thau system, targeting a gene coding thaumatin-like protein, detected almond among eight species, but cross-reactivity with apricot was observed. AlmondITS was designed on the ITS sequenced region, an internal transcribed spacer from ribosomal RNAs, and its specificity was determined in a study with 35 stone fruits (including hazelnut and olive) and 214 commercial products. Among the species and foods selected for the mentioned specificity studies, olive has not been included, with the exception of the ITS system, and none of them have used vegetable oils as a food matrix.

The specificity of each primer pair (reported to be used in probe-based qPCR assays) was assessed by qPCR protocols using SYBR Green I chemistry. We considered using the SYBR Green I chemistry because this dye is less expensive, less time consuming, and has easier optimization steps than the probes chemistry. This dye binds to dsDNA and can be used to detect and quantify the amplification products during PCR by monitoring overall fluorescence emission. However, the major disadvantage is that SYBR Green binds nonspecifically to double helix and this may involve a loss of specificity if primers-dimers or nonspecific fragments are present [64]. Therefore, the evaluation of the specific dissociation curves and melting temperatures are essential to differentiate specific amplicons from other non-specific products.

The selected primer sets were analyzed to determine those that allowed differentiation between olive and the adulterant species (almond or hazelnut). The results obtained showed that all systems amplified both the target species (almond or hazelnut) and olive. Moreover, the systems Hsp1, Cora1FW/RS and Cora13 for hazelnut, and Pdr6 and Pru du 1 for almond showed overlapping melting curves with olive (Table 1), so they were discarded. However, the difference in the Tm value between the amplicon of hazelnut and olive was 7.5 °C and 4.5 °C for Nocc1 and Cora1F2/R2, respectively, and 5.5 °C, 7.0 °C, and 7.5 °C for the amplification of almond and olive by Mad1, thau, and AlmondITS (Table 1).

**Figure 1:**
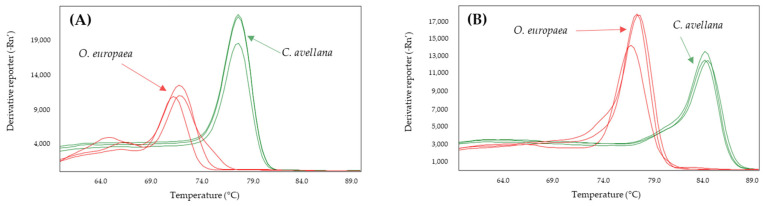
Derivates of melting curves for olive (red) and hazelnut (green) DNA obtained by SYBR-qPCR of Cora1F2/R2 (**A**) and Nocc1 (**B**) systems assayed in triplicate. Annealing temperature qPCR: 60 °C.

**Figure 2:**
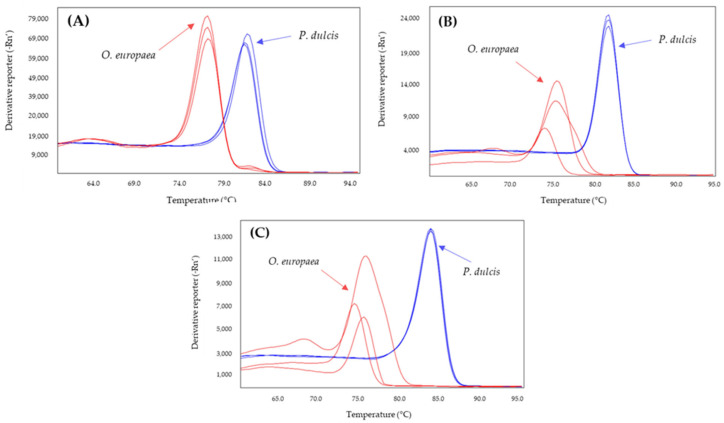
Derivates of melting curves for olive (red) and almond (blue) DNA obtained by SYBR-qPCR of Madl (**A**), thau (**B**), and AlmondITS (**C**) systems assayed in triplicate. Annealing temperature qPCR: 61 °C.

The effect of increasing the annealing temperature of the systems to 61, 63, and 65 °C was analyzed to determine whether the specificity of the systems increased (Table 2). From these results, the annealing temperatures were selected to achieve the greatest difference between the Tm values of the adulterant species and that of the olive: 60 °C for Nocc1 and Cora1F2/R2 and 61 °C for Madl, thau, and AlmondITS. In the melting curves obtained, the olive was clearly differentiated from the possible adulterating species, hazelnut (Figure 1) or almond (Figure 2).

**Figure 3:**
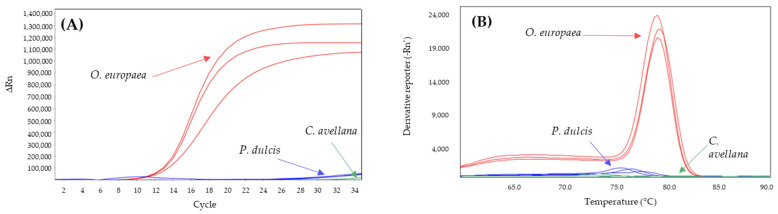
Amplification curves (**A**) and derivate of melting curves (**B**) of D-trnL SYBR-qPCR for olive (red), hazelnut (green), and almond (blue) assayed in triplicate.

On the other hand, the D-trnL system was also analyzed for its specificity toward hazelnut and almond, under the conditions described in Alonso-Rebollo et al. [45]. The results showed how it specifically amplifies olive with a Cq of 11.3 ± 0.1 and a melting peak at 79.1 ± 0.1 °C, while hazelnut did not amplify, and almond amplified from cycle 30 onwards with a melting peak at 76.6 ± 1.1 °C (Figure 3). Therefore, it was considered as an olive oil specific system.

Besides, the selected systems not only have to allow discrimination between species but also have to show, within a wide linear dynamic range, high sensitivity and be efficient. In this sense, the analysis of the calibration curves is the main parameter used to determine the amplification efficiency and the adjustment coefficient (R^2^). The statistical analysis of the Cq values allowed us to determine the sensitivity of the systems to quantify (LOQ) and to detect (LOD) (Table 3).

Among the five systems analyzed (Table 3), only the ITS system confirmed the detection ability of almond but not its quantification capacity under the tested conditions. This is a system whose previous validation included a Taq-Man probe [62], which suggests that this probe is a requirement to allow quantification. The thau system, although it allowed the quantification of almond DNA, is the one that showed a range of linearity and its LOQ was established between 50 ng and 50 pg of DNA, increasing the lower detection threshold to 5 pg of DNA per reaction. The Nocc1 system allowed to quantify between 500 ng and 50 pg and specifically detect up to 5 pg of DNA from hazelnut. The Madl systems, specific for almonds, and Cora1F2/R2, specific for hazelnuts, turned out to be the systems that showed the highest LOD, up to 5 pg, for each species, also extending the LOQ up to the same amount.

The systems Madl, Cora1F2/R2, and D-trnL showed correlation coefficients greater than 0.995 and qPCR efficiencies between 97 and 100% (Table 3). Both, the correlation coefficients and the qPCR efficiencies are similar to those described by these systems for almond (0.996 and 98.0%) [54] and olive (0.999 and 105.0%) [45], in the absence of comparative data for hazelnut, which shows that they are systems with high efficiency and a wide range of detection.

Before applying these systems to DNA isolated from vegetable oils, the robustness of the almond (Madl) and hazelnut (CoraF2/R2) specific systems was tested on two different qPCR instruments (QuantStudio TM5 and iCycler iCycler iQ™) and three different master mixes (SYBR-EURX, RealQ Plus-AMPLIQON and KAPA SYBR^®^-KAPA BIOSYS-TEMS). The results obtained showed that the melting profiles obtained on the iCycler iQ™ qPCR instrument clearly discriminated the target samples from the adulterant samples; however, in both cases, a ± 1 °C shift in the melting peaks of the target species was observed, although the melting temperatures of the adulterants were not altered. Furthermore, when using different SYBR-based products, no significant differences (*p* < 0.05) were observed in the melting temperatures obtained for Madl (almond: 81.8 ± 0.19, olive: 77.28 ± 1.07) and CoraF2/R2 (hazelnut: 79.13 ± 0.22, olive: 74.39 ± 0.4) systems.

Thus, the parameters are in good agreement with the acceptance criteria defined for qPCR method performance [65], so they were selected to determine their ability to specifically detect almond, hazelnut, and olive DNA, respectively, in mixtures of DNA extracted from vegetable oils.

### 2.3. Sensitivity in DNA Isolated from Vegetable Oils

In order to determine the capacity of each system to detect possible adulterations in vegetable oils, DNA was extracted from almond, hazelnut, and olive oils and binary mixtures of these DNAs were made in nine different percentages ranging from 100:0% to 0:100% (olive:adulterant). To detect the adulterant species, the sensitivity and specificity of the systems selected were analyzed by qPCR. The evaluation of specific dissociation curves and melting temperatures turns critical for the discrimination of specific amplicons and other unspecific products [60]. In this sense, considering the different dissociation patterns presented by the systems under study, the sensitivity analysis in mixtures of DNA from oils was carried out based on the melting profiles generated in each system.

**Figure 4:**
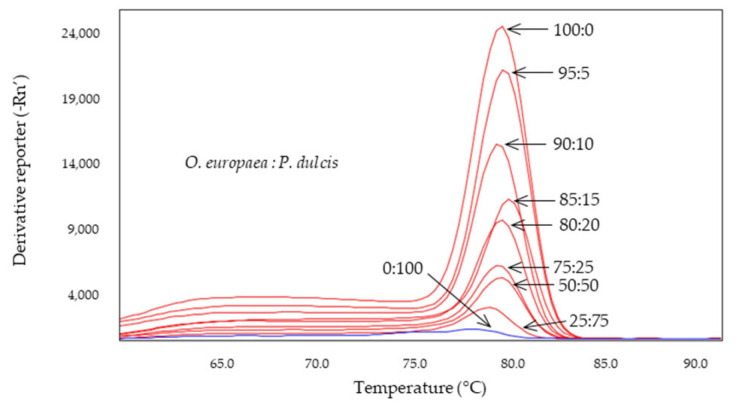
Derived from melting curves of D-trnL obtained by SYBR-qPCR in binary mixtures at different percentages of DNA obtained from olive:almond oils. The percentages are shown in the figure.

The D-trnL system was found to be specific for olive allowing the detection of olive in up to a 5% mixture in 95% of almond DNA (Figure 4) without detecting almond or hazelnut DNA. The specificity of this system, already described in Alonso-Rebollo et al. [45] against maize, sunflower, peanut, coconut, and rapeseed, is now extended to almond and hazelnut. Furthermore, the results obtained in this work suggested that this system can be applied not only to types of olive oil (extra virgin, virgin, olive oil, and pomace oil) [45], but it can also be used to detect the presence of olive in binary mixtures with only 5% olive DNA. Therefore, this system has potential as a tool for the specific detection of olive oil against the main vegetable oils on the market. This system could be used as an olive oil authentication system and could also be included in studies of possible fraud due to adulteration, allowing the establishment of a positive control for olive oil.

**Figure 5:**
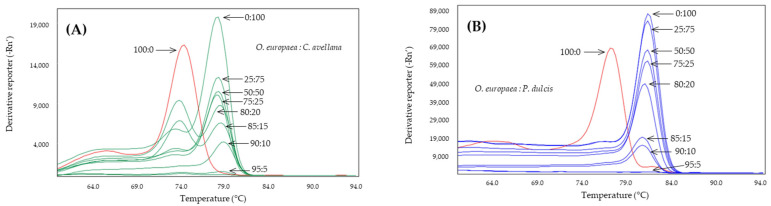
Derived from melting curves of Cora1F2/R2 (**A**) and Madl (**B**) obtained by SYBR-qPCR in binary mixtures at different percentages of DNA obtained from olive:adulterant oils: hazelnut (green) or almond (blue). The percentages are shown in the figure which includes 100% olive oil (red).

The Cora1F2/R2 system, designed to detect hazelnut, has also been studied in binary mixtures with olive DNA at different percentages (Figure 5A). The results showed how the system is able to specifically detect hazelnut in these mixtures up to a lower threshold of 10%, not detecting hazelnut in the 95 and 100% olive mixture. In this case, it was observed how, as the presence of olive DNA increased, a second melting peak appeared at 74.4 ± 0.5 °C, compared to the corresponding almond peak of 78.9 ± 0.1 °C. A sample of olive DNA extracted from leaves at a concentration of 100 ng was included in the assay, which yielded a melting peak at 74.5 ± 0.1 °C, coinciding with the second peak observed in the mixtures with 15 and 20% hazelnut DNA.

The system selected to specifically detect almond trees, Madl, was tested under the same conditions as the previous systems. The results (Figure 5B) showed that this system was able to detect up to 10% almond DNA in the presence of olive DNA. In this case, as with D-trnL, the detection resulted in unique, almond-specific melting peaks at Tm of 81.6 ± 0.1 °C. At percentages lower than 10%, no amplification was obtained. The olive positive control showed its characteristic melting peak at 77.3 ± 0.1 °C but no secondary peaks were detected at this temperature in the mixtures at different percentages, probably due to the competitiveness of the target.

The overall results allow us to establish Cora1F2/R2 as systems that can detect the presence of 5% hazelnut and 10% almond in olive oils, respectively. Likewise, the D-trnL system can be used to specifically detect olive in oils, so it can be used as an amplifiability control. However, the presence of almonds or hazelnuts in olive oils as adulterants can be found in lower percentages and can cause health problems, not to mention the food fraud that it entails and the food safety problem that it constitutes.

### 2.4. Detection of Almond and Hazelnut in Olive Oils

In order to increase the sensitivity of the almond and hazelnut detection systems, a PCR pre-amplification process was carried out followed by a second amplification phase using qPCR. This nested PCR has been proposed by other authors as an effective approach to detect DNA targets as almonds or hazelnut [30,58] or peanut [66] at trace levels.

**Figure 6:**
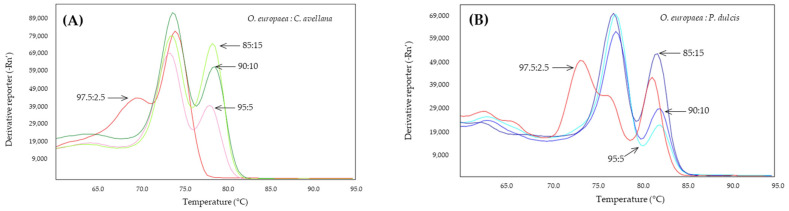
Derivates of melting curves of Cora1F2/R2 (**A**) and Madl (**B**) obtained by SYBR-qPCR on DNA obtained from binary mixtures of oils at low percentages (2.5–15%) of adulterant: hazelnut (green) or almond (blue). The percentages are shown in the figure.

The nested PCR was used to analyze the DNA extracted from adulterated olive oils in percentages established in the laboratory of 15, 10, 5, and 2.5% of almond or hazelnut oils (Figure 6). In order to amplify all the DNA recovered from the extraction process, first-stage pre-amplification was performed at 58 °C and to reduce non-specific amplifications, a DNA polymerase proof-reading was used. After pre-amplification by PCR, 10 µL of the product was used for second-stage qPCR, according to the most optimal conditions described previously in this work.

In the case of mixtures of hazelnut oil in olive oil at low percentages, it was possible to detect the presence of hazelnut DNA in percentages of 15, 10, and 5% with the presence of two well-defined peaks, with a slight displacement of both with respect to those observed in the simple qPCR analyses at 78.5 and 74 °C (Figure 6A). This slight displacement of the melting peaks has also been observed in the DNA obtained from different oils with respect to the DNA obtained from seeds [47]. At a percentage of 2.5% of hazelnut oil in olive oil, the system only detects olive DNA, and another peak of lower fluorescence is generated, probably due to non-specific amplification.

Despite the existence of multiple methodologies for the detection of minor adulterants in olive oils [6], the detection of hazelnut oil is especially complex due to the similarities of both oils in terms of lipid profile. Systems based on a chemometric analysis of the NIR (near-infrared spectroscopy) spectra with PCA (principal component analysis) processing [67], and on mass spectrometry combined with multivariate regression techniques [68], have been described as hazelnut detection techniques in olive oils. These methods are the most sensitive up to now but require precision equipment accompanied by complex statistical analysis. A different approach, based on DNA, has demonstrated its ability to detect up to 10% hazelnut in olive oils [47]. These DNA-based methods use less time and costs, being an optimal alternative to achieve this objective.

The Madl system also made it possible to detect almond by nested qPCR in olive oil adulterated with 2.5% almond oil (Figure 6B). At this last percentage, a third peak appears at 74 °C, probably due to the decrease in the annealing temperature during the pre-amplification phase, which has favored the appearance of this non-specific product in the absence of target DNA in the same way that it has produced the appearance of the peaks corresponding to olive, which did not appear in the simple qPCR assays.

The usefulness of the nested-PCR and its potentiality in the detection of diverse allergens has been highlighted previously [58], now its applicability in the detection of adulterants in oils with allergenic potential is emphasized. The presence of proteins and peptides in cold-pressed oils is less commonly discussed, though they are an important component that may influence the oils’ stability and also cause an allergenic response in sensitive consumers [69,70] and allergenic proteins have been detected in this type of oil [71].

The Cora1F2/R2 and Madl systems have been previously correlated with the ELISA (enzyme linked immunosorbent assay) method in the detection of hazelnut and almond in food, respectively [54,60]. Both works suggest the possibility of applying, in the first instance, qPCR as a screening method to carry out a confirmation process using ELISA to obtain complete and reliable information in complex matrices in which allergens can be masked [60].

In summary, with this work, we have been able to describe the application of qPCR amplification systems based on SYBR-Green for the detection of almond and hazelnut as olive oil adulterants and a control system for olive oil with high sensitivity and specificity against both adulterant species. The application in adulterated olive oils demonstrated the ability of these systems to detect up to 5% adulteration with hazelnut and up to 2.5% with almonds by means of nested-PCR, thus defining a time and cost-efficient tool for the identification of both nut adulterants in olive oils.

## 3. Materials and Methods

### 3.1. Plant Tissue and Oil Samples

Almond and hazelnut samples were collected from wild tress locally known as almond (*P. dulcis)* and hazelnut (*C. avellana*) in the locality of Villasidro (Burgos, Spain, GPS coordinates: 42°26′14″ N and 4°4′21″ W). Olive (*O. europaea*) samples were taken from an olive mill located in Ahigal de los Aceiteros (Salamanca, Spain, GPS coordinates: 40°52′31″ N and 6°44′52″ O). All samples consisted of fresh, healthy leaves taken from different trees and were stored at −80 °C until DNA isolation.

Olive oil, produced from olives of the Picual variety, was purchased from olive oil mills, and almond and hazelnut oils were obtained from local grocery retailers. The labeling of all oils describes them as virgin oils from a single plant species.

### 3.2. DNA Extraction

The frozen leaf was ground into fine powder in the presence of liquid nitrogen with a porcelain mortar and pestle. In total, 100 mg of the fine power were transferred into a 0.5 mL tubes for DNA extraction. Two methods were assayed to isolate DNA from vegetable tissue: the protocol described by Doyle and Doyle [50], and an adaptation of it for olive trees and other oilseed species reported by Alonso-Rebollo et al. [45].

DNA was extracted from oil samples according to the protocol developed by Ramos-Gómez et al. [44] for vegetable oils, with minor modification. In detail, 1 mL of mixtures of oils was mixed with 0.5 volumes of pre-warmed lysis buffer (2% (*w*/*v*) CTAB, 1.4 M NaCl, 50 µM dithiothreitol (DTT), 20 mM ethylenediaminetetraacetic acid (EDTA), 100 mM trishydroxymethyl amino methane (Tris)–HCl, pH 8.0, 2% (*v*/*v*) Tween-20, 20 μg Proteinase K), and 0.5 volumes of hexane at 65 °C for 30 min. Subsequently, 500 µL of cold chloroform:isoamyl alcohol (24:1) were added and mixed for 2 min and centrifuged during 15 min at 20,000× *g* (4 °C). This step was repeated in order to purify the sample. For DNA precipitation, the aqueous phase was transferred to a new tube and 0.8 volumes of cold isopropanol were added. The tubes were kept at −20 °C for 1 h and centrifuged at 20,000× *g* (4 °C) for 30 min. Then, the DNA pellet was washed with 70% (*v*/*v*) ethanol. Finally, the DNA pellet was resuspended in 12.5 µL of 0.1 × TE buffer (10 mM Tris, 1 mM EDTA).

The quantity and quality of the isolated DNA was determined by measuring the absorbance at 260 nm and the 260/280 nm ratio using a UV spectrophotometer (PowerWave XS2, BioTek Instruments, Inc., Saint Quentin, France).

### 3.3. Oligonucleotide Primers

For the detection of almond and hazelnut, for each species, five systems described in the literature were selected based on their specificity against other species and their suitability for qPCR (Table 4).

The olive-specific system, D-trnL (Table 4), was previously designed by our research group for the authentication of olive oil [45]. This system, based on the intron region of the chloroplast tRNA *trnL* (UAA) gene, showed specificity among 7 oilseed species.

All the primers were synthetized by Metabion (Martinsried, Germany).

### 3.4. qPCR Assay

The systems were tested by SYBR Green-based quantitative PCR (qPCR). The assays were carried out in a mixture of 20 μL containing 1×SYBR Green Master Mix (EURx, Gdańsk, Poland), forward and reverse primers at the concentration previously described (Table 4). and 50 μg or 5 µL of DNA from foliar tissue or vegetal oils, respectively. The initial amplification conditions assayed were as previously reported for each system (Table 4). Moreover, in our study, the amplification program was followed by a melting cycle of 95 °C for 1 min and annealing at 60 °C for 3 min (for the correct formation of DNA duplexes), being followed by a melting curve (60 °C up to 95 °C) with temperature increments of 0.5 °C every 15 s.

The qPCR assays were performed in a fluorometric thermal cycler QuantSudioTM 5 Real-Time PCR System (Thermo Fisher Scientific, Waltham, MA, USA). All samples were analyzed in triplicate and no template control (NTC) was included in any assay to ensure the absence of contamination.

### 3.5. Evaluation and Selection of the qPCR Systems

The linear dynamic range of the systems was determined by performing a standard curve for each system. The standard curves were designed including three replicates of eight ten-fold dilutions of DNA from each species (from 500 ng to 0.5 pg). All systems included an NTC to detect DNA contamination. The efficiency (E = 10^(−1/slope)^ − 1) and the correlation coefficient (R^2^) were calculated from the equation of the curve for each system (Cq = a log [DNA] + b).

The sensitivity of the methods, based on the standard curve, was evaluated by determining the limit of detection (LOD), defined as the lowest concentration at which the analyte can reliably be detected with a probability of 95%, and the limit of quantification (LOQ), understood as the lowest concentration at which there is some confidence in the accuracy of the reported measurement. The statistical significance between the Cq values was evaluated by analysis of variance (ANOVA) with a significance level of 95% (*p* < 0.05) considering the three replicates of each point on the standard curve.

Once the optimal experimental conditions for each system were established, the robustness of the systems was evaluated based on the melting profiles obtained in two qPCR instruments: QuantSudioTM 5 Real-Time PCR System (Thermo Fisher Scientific, Waltham, MA, USA) and iCycler iQ™ Real-Time PCR Detection System (Bio-Rad, Hercules, CA, USA). Results obtained using different master mixes were also compared: 2× SYBR Green Master Mix (EURx, Gdańsk, Poland), RealQ Plus 2× Master Mix Green (AMPLIQON, Odense M, Denmark), and KAPA SYBR^®^ FAST Qpcr Master Mix (KAPA BIOSYSTEMS, Wilmington, MA, USA). All assays were performed in triplicate, including target and adulterant DNA.

### 3.6. Detection of Olive Oil Adulteration

For the detection of olive oil adulteration, different mixtures of DNA obtained from olive oil and almond oil, or hazelnut oil, were prepared at ratios of 100:0%, 95:5%, 90:10%, 85:15%, 80:20%, 75:25%, 50:50%, 25:75%, and 0:100%, respectively. In the assays for the adulteration of olive oil with almond and hazelnut, olive controls consisting of 100 ng of DNA from olive leaves per reaction were included. On the other hand, in the detection of olive in DNA mixtures of oils, DNA from almond leaves and hazelnut leaves were included as controls at 100 ng per reaction. The analysis of the results obtained allowed for the determination of the sensitivity of the specific systems of each of the species.

#### Nested qPCR

To improve the sensitivity of the systems at low percentages of adulteration, a nested qPCR was carried out with DNA obtained from a mixture of olive oil and almond oil or hazelnut at ratios of 97.5:2.5%, 95:5%, 90:10% and 85:15%, respectively.

The optimization process was based in the pre-amplification of the DNA by conventional PCR in a Veriti™ Thermal Cycler (Thermo Fisher Scientific, Waltham, MA, USA), with the selected systems and same conditions, during only 5 cycles at 58 °C of annealing temperature and using a proofreading DNA polymerase (PfuPlus! DNA Polymerase, EURx, Gdańsk, Poland). The PCR products were then amplified by qPCR, as described above.

## 4. Conclusions

The CoraF2/R2 and Madl systems have shown, through distinctive melting curves, adequate specificity for hazelnut and almond, respectively. The results showed that adulteration of up to 5% hazelnut or almond oils in olive oils can be detected by a single qPCR assay. The use of nested qPCR allowed to increase the sensitivity threshold in both systems, detecting up to 2.5% adulteration of olive oil with oils from these two adulterant species. In summary, the present research has shown that SYBR-based qPCR assays can be a rapid, precise and accurate method to detect adulteration of olive oil with almond or hazelnut.

## Figures and Tables

**Table 1:** Melting temperature (Tm) obtained for hazelnut (*C. avellana*), almond (*P. dulcis*), and olive (*O. europaea)* in SYBR-qPCR assays of the systems used in this study ^a^.

Adulterant Specie	System	Tm Amplicon (°C)	
		Adulterant	*O. europaea*
*C. avellana*	Hsp1	84.0	84.0
	Nocc1	84.5	77.0
	Cora1FW/RS	79.0	79.0
	Cora1F2/R2	80.0	75.5
	Cora13	86.0	86.0
*P. dulcis*	Madl	83.5	78.0
	Prd6	86.0	86.0
	Pru du 1	75.5	75.5
	thau	82.0	75.0
	AlmondITS	84.5	77.0

^a^ Annealing temperature qPCR: 60 °C.

**Table 2:** Melting temperature (Tm) for hazelnut (*C. avellana*), almond (*P. dulcis*), and olive (*O. europaea)* obtained at different annealing temperature ranging from 60 to 65 °C.

Adulterant Specie	System	Tm Amplicon (°C) ^a^							
		Adulterant				*O. europaea*			
		60 °C	61 °C	63 °C	65 °C	60 °C	61 °C	63 °C	65 °C
*C. avellana*	Nocc1	84.5	84.5	84.5	84.5	78.0	84.5	84.5	84.5
	Cora1F2/R2	79.0	79.0	79.0	N/A	74.5	78.0	80.0	80.0
*P. dulcis*	Madl	81.5	81.5	N/A	N/A	78.0	77.0	79.0	79.0
	thau	82.0	82.0	N/A	N/A	75.0	75.0	N/A	N/A
	AlmondITS	84.5	84.0	83.5	N/A	77.0	75.0	82.5	N/A

^a^ N/A: non-amplification.

**Table 3:** qPCR validation parameters obtained for the specific detection systems for almond and hazelnut selected in this study.

Target	System	Calibration Curve		Accuracy and Efficiency		Analytical Sensitivity	
		Slope	Intercept	R^2 a^	E ^b^ (%)	LOD ^c^ (pg)	LOQ ^d^ (pg)
*C. avellana*	Nocc1	−3.306	21.553	0.997	100.31	5	50
	Cora1F2/R2	−3.372	19.016	0.997	97.95	5	5
*P. dulcis*	Madl	−3.390	24.702	0.995	97.25	5	5
	thau	−3.255	25.247	0.977	102.89	5	50
	AlmondITS	−5.011	20.311	0.947	58.33	5	-
*O. europaea*	D-trnL	−3.322	14.499	0.999	100.01	0.5	0.5

^a^ Correlation coefficient; ^b^ PCR efficiency; ^c^ limit of detection; ^d^ limit of quantification.

**Table 4:** qPCR systems used in this study: their features and amplification conditions.

Name	Sequence (5′-3′)	PCR Conditions ^a^					Target (bp)	GenBank Accesion	Ref.
		Conc. (nM)	Den. t (s)	Ann. T (°C), t (s)	Ext. t (s)	Cycles			
Hsp1	F: AGCGTCGAGAGTGGCAAGTTC R: CCTGCTCGCCTCCGCTTTC	200	15	66, 45	60	50	126	AF021807	[58]
Nocc1	F: GGCAAGTTCGTGAGCAGGTTC R: CTTTCGGAATAGTCACAGTGAG	500	15	60, 60	--	35	100	AF021807	[59]
Cora1FW/RS	F: GCTTTGTCCGACAAACTGGAG R: TCCTATGGTGTGGTACTTGCTG	250	30	55, 30	40	35	105	Z72440	[60]
Cora1F2/R2	F: ACTACATAAAGCAAAAGGTTGAAG R: TCGTAATTGATTTTCTCCAGTTTG	800	15	60, 60	--	35	109	Z72440	[54]
Cora13	F: GCGGTCATCACAGTATCGCTT R: GTCACGTACCTGTAGATCCACGAC	200	15	60, 60	--	40	101	AY224599	[53]
Madl	F: CCTAGCGGAGGATCCATCATC R: GGTCTCAATGAGCTTGAAGAG	160	15	60, 60	60	45	129	BQ641046	[61]
Prd6	F: CCGCAGAACCAGTGCCAGCT R: CCCCGGCACACTGGAAGTCCT	300	15	65, 45	--	--	121	EU919663	[58]
Pru du 1	F: AGTGTATTGTGATTGGCTCCC R: AGTCTTTGGCTTGCATTTGG	200	15	60, 60	--	40	100	KC969088	[36]
thau	F: ACTGAGCACAACGGAATATC R: TAGGATGCCGTGCGTAGC	500	15	58, 30	30	55	113	EU424262	[62]
AlmondITS	F: CTAGCCGAACGACCCGAGA R: CCGAGATAAAGGGGACGAG	300F 900R	5	60, 30	--	55	76	HF969276	[63]
D-trnL	F: GGGCAATCCTGTAGCCAAA R: ACGCAGTCCACTCCATTTGT	100	30	53, 30	60	40	110	DQ131560	[45]

^a^ Abbreviations: conc.: primer concentration; den.: denaturation; ann.: annealing; ext.: extension; t: time; T: temperature.

## Data Availability

The data presented in this study are available on request from the corresponding author.
